# Essential Functions of the Transcription Factor Npas4 in Neural Circuit Development, Plasticity, and Diseases

**DOI:** 10.3389/fnins.2020.603373

**Published:** 2020-12-01

**Authors:** Jian Fu, Ouyang Guo, Zhihang Zhen, Junli Zhen

**Affiliations:** ^1^Department of Emergency Surgery, The Second Hospital of Hebei Medical University, Shijiazhuang, China; ^2^Department of Biology, Boston University, Boston, MA, United States; ^3^Department of Neurology, Key Laboratory of Neurology of Hebei Province, The Second Hospital of Hebei Medical University, Shijiazhuang, China

**Keywords:** Npas4, neural activity, synaptic activity, neuroprotection, excitatory–inhibitory balance

## Abstract

Signaling from the synapse to nucleus is mediated by the integration and propagation of both membrane potential changes (postsynaptic potentials) and intracellular second messenger cascades. The electrical propagation of postsynaptic potentials allows for rapid neural information processing, while propagating second messenger pathways link synaptic activity to the transcription of genes required for neuronal survival and adaptive changes (plasticity) underlying circuit formation and learning. The propagation of activity-induced calcium signals to the cell nucleus is a major synapse-to-nucleus communication pathway. Neuronal PAS domain protein 4 (Npas4) is a recently discovered calcium-dependent transcription factor that regulates the activation of genes involved in the homeostatic regulation of excitatory–inhibitory balance, which is critical for neural circuit formation, function, and ongoing plasticity, as well as for defense against diseases such as epilepsy. Here, we summarize recent findings on the neuroprotective functions of Npas4 and the potential of Npas4 as a therapeutic target for the treatment of acute and chronic diseases of the central nervous system.

## Introduction

Many adaptive changes in brain function such as learning depend on the capacity of individual neurons to transduce changes in postsynaptic membrane potential into longer-lasting changes in gene transcription, leading to stable biochemical and structural modifications (termed neural plasticity) (Fernandez-Albert et al., 2019). To achieve gene expression changes in response to specific synaptic inputs, intercellular second messenger cascades must activate transcription factors (TFs) able to reach the nucleus. One such pathway is the propagation of intracellular calcium transients that activate various kinase cascades and protein–protein associations leading to TF activation. Among these transduction molecules are the large family of highly conserved proteins containing the Per-Arnt-Sim (PAS) domain. These PAS proteins are implicated in numerous critical biological pathways, including neurological responses to external stimuli during development and adulthood. One mechanism vital to neural circuit development and adaptive changes is homeostatic regulation of the balance between excitatory and inhibitory synaptic transmission.

The neuronal PAS domain-containing protein 4 (Npas4), a member of the PAS family characterized by a conserved basic-helix-loop-helix motif and PAS domain (Greb-Markiewicz et al., 2018), acts as an inducible immediate early gene (IEG) activated with minutes of stimulation to regulate the formation of inhibitory synapses (Lin et al., 2008). Importantly, the physicochemical properties and reveals the neuro-modulatory role of Npas4 in crucial pathways involved in neuronal survival and neural signaling hemostasis (Fahim et al., 2018). Moreover, Npas4 differentially communicates increases in a neuron’s spiking output and synaptic inputs to the nucleus, enabling gene regulation to be tailored to the type of depolarizing activity along the somato-dendritic axis of a neuron (Brigidi et al., 2019). Activated Npas4 regulates the transcription of multiple genes, such as other transcription factors, channel proteins, G-protein signaling molecules, kinases, phosphatases, and genes involved in the modulation of synaptic functions via ubiquitination, trafficking, and receptor endocytosis. Through these changes, Npas4 controls the synaptoplastic changes underlying experience-dependent learning and memory in the striatum, hippocampus, cortex, and amygdala (Ploski et al., 2011; Guo et al., 2012; Hartzell et al., 2018; Heroux et al., 2018). In addition, several results demonstrated that Npas4 involves preoperative anxiety led to GABAergic system impairment in spinal cord and caused hyperalgesia (Wu et al., 2019).

Npas4 also regulates the development of glutamatergic and GABAergic synapses in neurons to maintain neurocircuit homeostasis essential for information processing and memory formation (Spiegel et al., 2014). Further, maintenance of the excitatory–inhibitory balance is critical for neuronal survival as excess excitation (excitotoxicity) is common mechanisms for cell death under various pathological conditions such as ischemia and epilepsy (Woitecki et al., 2016; Buchthal et al., 2018). Studies on the effects of cerebral ischemia (Choy et al., 2015) and neuronal activity (Sun and Lin, 2016) on Npas4 expression have been reviewed. Here we will further summarize the associated research progress on Npas4 functions in nervous system diseases.

### Npas4 Regulates Excitatory–Inhibitory Balance Within Neural Circuits

**FIGURE 1:**
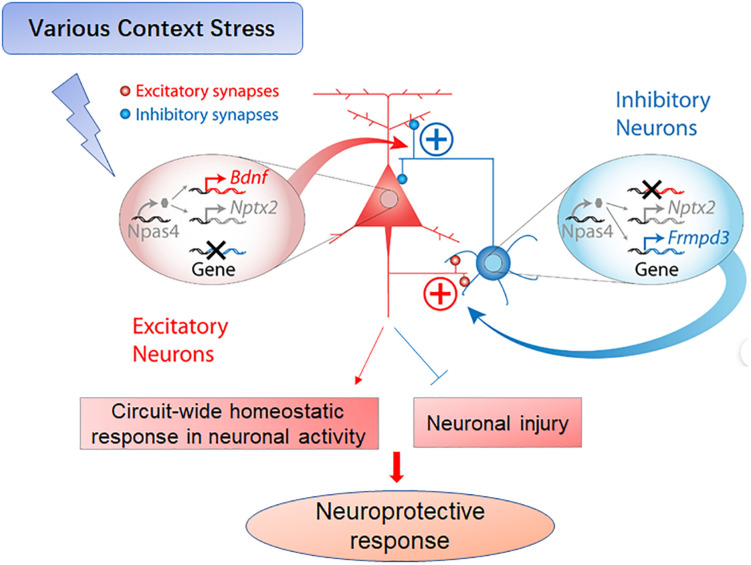
Schematic of Npas4-mediated neuroprotective functions following various stressors. As a transcription factor, Npas4 regulates the expression of a large number of downstream genes (e.g., Bdnf, Nptx2 and Frmpd3) that mediate the diverse effects of Npas4 on synapses. In excitatory neurons, Npas4 upregulates perisomatic inhibitory synapses and downregulates excitatory synapses. In inhibitory neurons, Npas4 may upregulate excitatory synapses in response to activity, thereby enhancing inhibitory control mechanisms within circuits such as feedback and feedforward inhibition. Collectively, Npas4 contributes to the circuit-wide homeostatic regulation of neuronal excitation (see the Part of Figure 7 in Spiegel, I., et al. Adapted from Spiegel et al. (2014). Npas4 regulates excitatory-inhibitory balance within neural circuits through cell-type-specific gene programs. *Cell* 157, 1216–1229).

Npas4 regulates excitatory–inhibitory balance by enhancing inhibitory synaptic transmission (Lin et al., 2008; Sun et al., 2020) through its actions as a transcription factor responsive to excitation-coupled postsynaptic calcium (Ca^2 +^) influx, thereby activating distinct sets of early- and late-response genes required for the formation of inhibitory synapses (Spiegel et al., 2014). Further, Npas4 induces distinct gene expression programs within excitatory and inhibitory neurons to achieve a circuit-wide homeostatic response (Figure 1). For instance, numerous recent studies have shown that Npas4 activation promotes the formation of inhibitory synapses in the developing visual system and adjusts the homeostatic balance in excitatory neurons to induce visual cortical plasticity as an adaptive response to sensory input (Maya-Vetencourt et al., 2012). Furthermore, Npas4 may act as a molecular switch to initiate homeostatic scaling in the hippocampus, a function that suggests new therapeutic approaches for the treatment of epilepsy (Shan et al., 2018). Collectively, these findings suggest that Npas4-mediated gene activation is critical for the regulation of the excitatory–inhibitory balance in multiple neural circuits (Hartzell et al., 2018; Heslin and Coutellier, 2018; Sharma et al., 2019).

Memories are believed to be encoded by distributed ensembles of neurons functionally coupled through synaptic potentiation, and the Npas4 gene has been identified as an important regulator of this associative synaptic plasticity (Rein et al., 2020). It was recently reported that Npas4 promotes memory discrimination by enhancing the inhibitory drive from local cholecystokinin (CCK)-expressing interneurons within these ensembles. This finding provides evidence for functional heterogeneity within the memory engram and reveals synaptic and circuit mechanisms used by ensembles to regulate the memory discrimination–generalization balance (Sun et al., 2020).

### Npas4 Regulates Experience-Induced Target Gene Programs in Neurons

Most commonly, Npas4 regulates gene transcription by binding to enhancers and promoters of target genes (Benatti et al., 2019). Notably, Ebert and Greenberg have reviewed that transcription factors c-fos and Egr1 activate genes for brain-derived neurotrophic factor (BDNF) and Arc to regulate synaptic development (Ebert and Greenberg, 2013). In cortical and hippocampal CA3 pyramidal neurons, Npas4 also induces BDNF expression, which increases the number of contacting inhibitory synapses, and thereby reduces excitability (Lin et al., 2008; Bloodgood et al., 2013). Strikingly, Npas4 overexpression in cultured neurons is sufficient to induce cell type-specific expression of target genes such as BDNF, Csrnp1, Frmpd3, and Rerg in the absence of other activity-regulating factors (Spiegel et al., 2014).

Another target of Npas4, the oncogenic E3 ubiquitin ligase gene murine double minute 2 (Mdm2), ubiquitinates and degrades postsynaptic density protein-95 (PSD-95) in rat hippocampal neurons, thereby reducing the strength of excitatory transmission (Colledge et al., 2003). Moreover, different subsets of Npas4 targets control the development of GABAergic synapses formed by distinct classes of interneurons, which provides a mechanism for independent regulation of inhibition received by subregions of a neuron (Lin et al., 2008). Notably, Npas4-mediated inhibition of Mdm2 expression may regulate dendritic spine formation in olfactory bulb granule cells following sensory experience, thereby modulating experience-dependent plasticity and olfactory discrimination learning (Yoshihara et al., 2014).

Npas4 also regulates the expression levels of genes that serve to increase excitatory input onto somatostatin (SST)-releasing neurons, such as Stac, Osgin2, Nptx2, Ppm1h, Bach2, and Gpr3, which in turn may enhance feedback inhibition within cortical circuits. Several Npas4 target genes in SST neurons are able to act at postsynaptic sites of excitatory synapses (Nptx2, Kcna1) or are homologous to related molecules (Frmpd3), suggesting that Npas4-regulated gene programs in SST neurons’ function is to promote the development of excitatory synaptic inputs and thus increase inhibitory drive within circuits (Spiegel et al., 2014). Although this action is distinct from that previously described in excitatory neurons (Lin et al., 2008), both would act control the excitatory–inhibitory balance in a highly cell type-specific manner.

In response to heightened neuronal activity, the aryl hydrocarbon receptor nuclear translocator 2 (Arnt2) protein recruits Npas4 to activity-dependent regulatory elements, where it induces the transcription of genes that increase somatic inhibitory input (Sharma et al., 2019). In addition, during embryonic development, knockdown of Npas4a genes, a homolog of Npas4 in zebrafish, results in a number of forebrain-specific defects including increased apoptosis and misexpression of the forebrain marker genes dlx1a and shha, suggesting a potential role for mammalian Npas4 in neurodevelopment (Klaric et al., 2014). Similarly, during contextual memory formation, Npas4 may also restrict the number of mossy fiber (MF)-CA3 synaptic contacts by upregulating Plk2 (Weng et al., 2018). In addition to various upstream kinase signaling pathways, Npas4 expression is also regulated by genes such as that encoding miR-142 in post-traumatic stress disorder (Ji et al., 2019) and by Hdac5 in the nucleus accumbens (NAc) to regulate reward-dependent conditioning (Taniguchi et al., 2017). These findings collectively highlight potential functions of Npas4 in the neurobehavioral stress response and in disorders associated with aberrant learning.

### Neuroprotective Functions of Npas4

It is well established that Npas4 expression regulates embryonic stem cells and early postnatal brain development by RE-1 silencing transcription factor (REST) and microRNAs (Bersten et al., 2014). In addition, Npas4 is also implicated in protection against several neurological disorders or downstream sequela, including cerebral ischemia and epilepsy, both of which are associated with altered excitatory–inhibitory balance. Moreover, Npas4 may regulate other downstream sequela of excitotoxicity, such as neuroinflammation or apoptosis (Rahim et al., 2018; Shan et al., 2018).

The increase in intrinsic activity during maturation of adult-born granule cells in the hippocampal dentate gyrus (DG), a simple cortical region that is an integral portion of the larger functional brain system called the hippocampal formation, is sufficient to alter the synaptic connectivity within hippocampal circuits, and Npas4 is required for activity-dependent spine development (Sim et al., 2013). Alternatively, decreased expression of Npas4 in CA3 may induce a decline in synaptic inhibition and impair contextual memory formation (Ramamoorthi et al., 2011). In zebrafish, Npas4 is also involved in contextual learning and establishment of GABAergic synapses in the hippocampus homolog, which is part of the brain social decision-making network (Teles et al., 2016).

As a recent achievement in this regard, it was reported that Npas4 controls neuronal homeostasis in epilepsy through the induction of Homer 1a (Shan et al., 2018), and Npas4 also as one of pivotal transcriptional target of Hdac3-mediated repression in neurodegenerative disease (Louis Sam Titus et al., 2019). Moreover, Npas4 expression was reported to decrease in parallel with the progression of Alzheimer’s disease (AD), particularly at Braak neurofibrillary tangle (NFT) stages I-II, corresponding to lesion development in transentorhinal/entorhinal cortex (Miyashita et al., 2014). Npas4 knockdown can mimics the effects of amyloid precursor protein (APP) deficiency on GAD65 activity and GABA levels. This finding suggests that Npas4 could be a critical signaling factor in APP-dependent inhibitory synaptic transmission and further implicates dysregulation of excitatory–inhibitory balance in AD pathogenesis (Opsomer et al., 2020).

The neuroprotective functions of Npas4 may also extend to drug-induced effects via regulation of drug-responsive genes. Repeated injections of the psychostimulant amphetamine (AMPH) into the NAc upregulate regional expression of Npas4, suggesting that Npas4 transcriptional activity mediates some of the neurological and behavioral effects of AMPH (Guo et al., 2012). Similarly, microarray analyses revealed that methamphetamine (METH) injection decreased the expression of Npas4 (Martin et al., 2012). In addition, Halawa et al. reported that nicotine prevented Npas4 expression in the locus coeruleus, which may reduce inhibitory synapse formation onto noradrenergic neurons (Halawa et al., 2018). While the molecular mechanisms underlying these changes and the precise effects on circuitry remain unclear, these changes in Npas4 expression concomitant with repeated drug administration suggest critical functions in the consolidation of addictive behaviors.

### Stress-Induced Changes in Npas4 Expression

Given that Npas4 possesses a unique arrangement of localization signals (Greb-Markiewicz et al., 2018) and has been implicated in neuroprotection against various form of stress, elucidating the factors driving neuron-specific expression in hippocampus, cortex, striatum, midbrain (Drgonova et al., 2016), amygdale (Ji et al., 2019; Cerqueira et al., 2020), and NAc (Taniguchi et al., 2017; Funahashi et al., 2019) may identify new therapeutic strategies for a host of central nervous system diseases.

Npas4 expression levels were reduced in mice and rats exposed to different forms of stress, including conditioned fear (Wang et al., 2018; Unno et al., 2020), sleep deprivation (Orozco-Solis et al., 2017), and anxiogenic environments (Jaehne et al., 2015). For instance, impaired neurogenesis and hippocampus-dependent fear memory in mice (Yun et al., 2010), contextual fear exposure in adolescent Long Evans rats (Heroux et al., 2018), and altered adult female behavior due to early maternal separation (Ryabushkina et al., 2020) have been reported to be associated with altered expression of Npas4. Npas4 knock-out mice also exhibited higher levels of the pro-inflammatory cytokines IL-6 and TNF-α post-stroke (Choy et al., 2016), and stroke-induced upregulation of Npas4 was found in brain regions linked to emotion and cognition (Leong et al., 2013). Additionally, regional alterations in Npas4 expression were found in the cerebral cortex of mice with genetic deletion of cadherin 13, a cell adhesion molecule that influences the development of brain circuits modulating addiction, locomotion, and cognition, such as circuits involving midbrain dopamine neurons (Drgonova et al., 2016). Most recently, Heslin and Coutellier reported that Npas4 deficiency combined with prenatal stress significantly impaired social recognition in mice (Heslin and Coutellier, 2018).

Collectively, these studies indicate pivotal contributions of Npas4 to long-term synaptic and circuit plasticity across multiple brain regions involved in fear, addiction, and spatial memory among other neural functions. In addition, these effects on circuit activity, especially modulation of excitatory–inhibitory balance, also confer protection against a variety of insults in animal models of disease.

## Conclusion

Npas4 is a recently discovered transcription factor that links neuronal activity to adaptive changes in circuit function, as well as stress to protective responses through modulation of excitatory–inhibitory balance (Figure 1). These adaptive changes have been implicated in neuronal survival, neural circuit plasticity, neurogenesis, and memory formation. Npas4 deficiency increases the susceptibility to neuronal damage from a variety of insults, suggesting Npas4 modulation as a potential therapeutic strategy against multiple conditions such as learning deficits, addiction, stroke, and AD. However, the molecular details of Npas4-dependent transduction and protective mechanisms are still unknown, as are the maintenance mechanisms for long-term changes and the precise effects on neural circuit activity.

Among the IEGs, Npas4 possesses a host of unique structural and functional features. It is expressed only in neurons, is activated selectively by neuronal activity, and is critical for sustaining normal circuit activity by orchestrating distinct gene expression programs in different neuronal populations, for review Sun and Lin (2016). While much additional research is needed to better understand how activity-dependent regulation of downstream genes influences neuronal survival, circuit activity, and behavior, it is reasonable to speculate that upregulation of Npas4 and associated signaling pathways can provide a novel strategy for the treatment of various neuropsychiatric and neurodegenerative diseases.

## Author Contributions

All authors listed have made a substantial, direct and intellectual contribution to the work, and approved it for publication. JF and JZ retrieved most of the sources and drafted the initial version of the manuscript.

## Conflict of Interest

The authors declare that the research was conducted in the absence of any commercial or financial relationships that could be construed as a potential conflict of interest.
